# LncRNA H19 drives atherosclerosis progression via the miR-let-7a/ITGB3 axis

**DOI:** 10.3389/fphys.2026.1801950

**Published:** 2026-05-20

**Authors:** Zi-Yang Hu, Yuan Li, Long-Yu Liu, Xin-Yu Chen, Kai-Xuan Lin, Abdallah Iddy Chaurembo, Xue-Shan Li, Guo-Qiang Huang, Xiao-Qian Wu, Zi-Feng Huang, Na Xing, Han-Bin Lin

**Affiliations:** 1 Zhongshan Hospital of Traditional Chinese Medicine Affiliated to Guangzhou University of Chinese Medicine, Zhongshan, Guangdong, China; 2 Guangzhou University of Chinese Medicine, Guangzhou, Guangdong, China; 3 Zhongshan Institute for Drug Discovery, Shanghai Institute of Materia Medica, Chinese Academy of Sciences, Zhongshan Guangdong, China; 4 School of Chinese Materia Medica, Nanjing University of Chinese Medicine, Nanjing, Jiangsu, China; 5 Guangzhou Municipal and Guangdong Provincial Key Laboratory of Molecular Target & Clinical Pharmacology, the NMPA and State Key Laboratory of Respiratory Disease, School of Pharmaceutical Sciences, Guangzhou Medical University, Guangzhou, China; 6 State Key Laboratory of Chemical Biology, Shanghai Institute of Materia Medica, Chinese Academy of Sciences, Shanghai, China; 7 School of Pharmacy, State Key Laboratory of Quality Research in Chinese Medicine, Laboratory of Drug Discovery from Natural Resources and Industrialization, Macau University of Science and Technology, Macao, Macao SAR, China

**Keywords:** adhesion molecules, atherosclerosis, inflammation, lncRNA H19, miR-let-7a

## Abstract

**Background:**

Atherosclerosis (AS) is a chronic inflammatory disease and a leading cause of global morbidity and mortality. Dysregulated expression of the long non-coding RNA (lncRNA) H19 has been implicated in AS progression. However, the underlying molecular mechanisms remain unclear.

**Methods:**

To determine the role of H19 in AS, the AS mouse model was established using ApoE^-/-^ mice fed a high-fat diet, and the AS cell model was generated by stimulating human umbilical vein endothelial cells (HUVECs) with oxidized low-density lipoprotein (oxLDL). H19 expression levels were subsequently measured. To investigate the underlying mechanism, H19 siRNA and was transfected into cells, and adeno-associated virus (AAV) expressing short hairpin RNA targeting H19 (AAV-sh-H19) was administered to mice to evaluate the functional impact of H19 on AS.

**Result:**

H19 was markedly upregulated in the aortae of AS mice and in the AS cell model, which correlated with enhanced adhesion molecule expression and systemic cytokine release. Mechanistically, H19 functioned as a molecular sponge for microRNA let-7a (miR-let-7a), thereby relieving the repression of its target integrin subunit beta 3 (ITGB3) and amplifying VCAM-1/ICAM-1/N-cadherin-dependent leukocyte recruitment. Luciferase reporter assays confirmed that miR-let-7a is a direct target of H19. Moreover, H19 knockdown increased miR-let-7a levels, thereby inhibiting endothelial cell adhesion and inflammatory responses. Conversely, treatment of mice with AAV-sh-H19 markedly attenuated AS lesion formation.

**Conclusion:**

These findings indicate that the H19/miR-let-7a/ITGB3 axis constitutes a targetable inflammatory checkpoint that links endothelial dysfunction to plaque initiation, highlighting H19 inhibition as a potential therapeutic target.

## Introduction

Atherosclerosis (AS) is a chronic, progressive inflammatory disease that primarily affects the arterial intima. It is characterized by the accumulation of lipid-rich plaques, primarily composed of free cholesterol and cholesteryl esters, within the intimal layer of medium- and large-sized arteries. The pathogenesis of AS initiates with endothelial dysfunction, which facilitates the subendothelial retention of low-density lipoprotein (LDL) particles and triggers a cascade of proinflammatory responses. Over time, this pathological process leads to luminal narrowing, increased arterial stiffness, and reduced vascular compliance, collectively heightening the risk of major adverse cardiovascular events (MACE), including myocardial infarction, ischemic stroke, and sudden cardiac death (Björkegren and Lusis, 2022). Although lipid-lowering therapies have markedly improved clinical outcomes, substantial residual cardiovascular risk persists. This underscores the urgent need to uncover novel molecular mechanisms underlying AS pathogenesis that extend beyond the traditional lipid-centric pathways.

AS progression involves endothelial dysfunction, lipid deposition, oxidative stress, and chronic immune activation. These processes drive monocyte recruitment, macrophage differentiation, oxidized LDL (oxLDL) uptake, and smooth muscle cell proliferation, leading to unstable plaques and thrombotic risk (Tasouli-Drakou et al., 2025). In this context, long non-coding RNAs (lncRNAs) are pivotal regulators of this pathology, modulating gene expression via chromatin remodeling and transcriptional control (Janczi et al., 2025). Within the AS microenvironment, lncRNAs govern cell proliferation (Li et al., 2018), apoptosis (Chen et al., 2017), inflammatory signaling (Li et al., 2024), and specifically regulating macrophage polarization (Ding et al., 2024). Among these, H19 has garnered significant attention for its involvement in cardiovascular diseases. Aberrant expression of H19 is linked to endothelial dysfunction and vascular inflammation (Hofmann et al., 2019). MicroRNAs (miRNAs) represent another class of non-coding RNAs, they are short transcripts of approximately 20–24 nucleotides that regulate gene expression by binding target mRNAs 3′ untranslated regions (3′UTRs) to induce degradation or translational repression (Catalanotto et al., 2016). In AS, miRNAs govern lipid metabolism (Ramírez et al., 2013), endothelial barrier integrity (Ashraf et al., 2025), and inflammatory signaling (Yang et al., 2015). Importantly, lncRNAs function as competitive endogenous RNAs (ceRNAs) by sequestering miRNAs, thereby derepressing target genes, a mechanism increasingly recognized in vascular biology (Rampin et al., 2024). For instance, an *in vitro* study demonstrated that H19 sponges microRNA let-7a (miR-let-7a), promoting cyclin D1 expression in vascular smooth muscle cells (VSMC). This interaction drives excessive VSMC proliferation and induces vascular remodeling (Sun et al., 2019). Another *in vitro* study focusing on the H19/miR-let-7a axis in AS showed that H19 inhibits miR-let-7a, leading to increased periostin expression. This upregulation induces inflammation, apoptosis and oxidative stress in human umbilical vein endothelial cells (HUVECs), effects that were reversed by H19 knockdown (Cao et al., 2019). Consequently, although the interplay between H19, miR-let-7a, and vascular function has been preliminarily verified, systematic *in vivo* investigations remain scarce. Furthermore, the target diversity of miR-let-7a leaves a critical gap in our understanding of their physiological relevance. Integrin subunit beta 3 (ITGB3) is established as a pivotal mediator of cell adhesion to ECM substrates and a driver of endothelial-leukocyte interactions (Ni et al., 2015; Li et al., 2024). While the regulation of ITGB3 by miR-let-7a is documented in the context of blastocyst implantation (Cheong et al., 2012), its specific contribution to the pathogenesis of AS remains unexplored. Given that endothelial cells (EC) are the primary cell type affected (Gimbrone and García-Cardeña, 2016), this study focuses on EC to bridge these distinct lines of evidence and elucidates the lncRNA-mediated regulatory network in AS. Specifically, this study aim to determine whether the miR-let-7a/ITGB3 axis serves as a key molecular driver of plaque progression, thereby positioning upstream lncRNAs as critical modulators of vascular pathology.

Endothelial dysfunction, the initiating event in AS, involves impaired vasodilation and increased permeability, promoting LDL infiltration and leukocyte transmigration (Qian et al., 2025). Activated EC upregulate adhesion molecules, such as vascular cell adhesion molecule-1 (VCAM-1), intercellular adhesion molecule-1 (ICAM-1), and N-cadherin, thereby enhancing monocyte recruitment and creating a pro-inflammatory microenvironment (de Souza et al., 2022). This dynamic crosstalk between dysfunctional endothelium and immune cells establishes a self-sustaining loop driving lesion progression. This response is amplified by adaptive immunity. Clinically, patients with AS exhibit a distinct CD4^+^ T cell subset, with T cells showing signs of activation and differentiation. Moreover, within AS plaques from these patients, both T cells and macrophages show elevated inflammatory factors (Kong et al., 2022). In summary, endothelial dysfunction triggers adhesion molecule expression, accelerating immune infiltration and vascular inflammation.

This study aimed to elucidate the precise molecular mechanisms by which H19 drives AS progression. This study demonstrated that H19 acts as a sponge for miR-let-7a, thereby upregulate ITGB3. This upregulation triggers the overexpression of endothelial adhesion molecules and amplifies pro-inflammatory signaling. Notably, our systematic *in vitro* and *in vivo* investigations not only provide comprehensive physiological evidence but also constitute the first validation of ITGB3’s critical role in AS pathogenesis. Our findings define the H19/miR-let-7a/ITGB3 axis as a critical driver of AS pathology and propose H19 inhibition as a viable therapeutic strategy.

## Materials and methods

### AS mouse model induction

All animal experiments were approved by the Institutional Animal Care and Use Committee of the Zhongshan Institute for Drug Discovery and were conducted in strict accordance with the relevant guidelines and regulations. Male wild-type C57BL/6J mice and *ApoE^-/-^* mice (C57BL/6J background; body weight: 22 ± 3 g; age, 6 weeks) were obtained from Shanghai Model Organisms Center, Inc. (Shanghai, China). Mice were housed under SPF conditions with a 12-hours light/dark cycle, temperature maintained at 22 ± 3°C, and relative humidity controlled at 55 ± 15%. Food and water were provided unrestrictedly. Following 1 week of adaptive feeding, *ApoE^-/-^* mice were fed a high-fat diet containing 1.25% cholesterol and 40% fat (Jiangsu Xietong, China) for 10 weeks to induce AS.

### Adeno-associated virus transduction

One week after arrival, the mice were randomly assigned to different groups and received tail vein injections of AAV serotype 9 (AAV9, Puhou Gene, Guangzhou, China). Two constructs were used: AAV-sh-NC and AAV-sh-H19, with the shRNA sequence for H19 as follows: 5’-GCAGTCATCCAGCCTTCTT-3’. The viral titer was 1.0×10 (Yang et al., 2015) vg/mL, and each mouse received a single intravenous injection of 100 μL of the viral suspension.

### Carotid artery ultrasound imaging

At the end of the 10 weeks high-fat diet feeding period, vascular hemodynamics and vessel wall thickness were assessed using a Vevo 3100 animal ultrasound imaging system (FUJIFILM VisualSonics, Canada). Prior to imaging, depilatory cream was applied to the neck and upper chest of each mouse and removed with tissue paper after 2 min. The mice were then anesthetized in an induction chamber with isoflurane in oxygen and subsequently positioned supine on a heated imaging platform equipped with electrocardiogram electrodes. Conductive gel was applied to the electrodes, and the paws were secured with 3M tape to ensure stable electrocardiogram (ECG) monitoring. Anesthesia was maintained using a nose cone to deliver isoflurane. The core body temperature was maintained using a feedback-controlled heating pad, and the respiratory rate was monitored throughout the experiment. Imaging was performed only when the heart rate stabilized within the physiological range of 400–650 beats per minute.

### Serum biochemical analysis

The mice were anesthetized, and whole blood were taken from the retro-orbital sinus plexus of mice under 1.5% isoflurane anesthesia. Serum was separated by centrifugation at 3,000 rpm for 15 min at 4 °C. Total cholesterol (TC), triglyceride (TG), low-density lipoprotein cholesterol (LDL-C), high-density lipoprotein cholesterol (HDL-C), aspartate aminotransferase (AST), and alanine aminotransferase (ALT) levels were measured using an automated biochemical analyzer (Hitachi, Japan) with commercial assay kits (Kehua Bio-Engineering Co., Ltd., Shanghai, China), following the manufacturer’s instructions.

### Histological evaluation of AS lesions in the aortic root

The mice were sacrificed, and the hearts along with the aortas were rapidly excised, rinsed in ice-cold PBS, and embedded in OCT gel. Cryosections (10 μm thick) were prepared using a cryostat microtome and were mounted on glass slides. Sections were stained with Oil Red O (Solarbio, Beijing, China), hematoxylin and eosin (HE; Solarbio, Beijing, China), and Masson’s trichrome (Solarbio, Beijing, China) according to the manufacturer’s protocol. Quantitative analysis of the lesion area and composition was performed using ImageJ software.

### Cell culture

HUVECs (#PSC-01) and RAW 264.7 cells (#SCSP-5036) were purchased from the Cell Bank of the Chinese Academy of Sciences (Shanghai, China). Mouse aortic vascular smooth muscle cells (MOVAS, #FH1289) were purchased from Fuheng Biology (Shanghai, China). HUVECs were cultured in endothelial cell medium (ECM; ScienCell, USA) supplemented with 5% fetal bovine serum (FBS), 1% penicillin-streptomycin, and 1% endothelial cell growth supplement (ECGS). Cell passage numbers were calculated from the time of laboratory receipt, and experiments were conducted using cells from passages 1 to 6. MOVAS and RAW 264.7 cells were cultured in DMEM supplemented with 10% FBS and 1% penicillin-streptomycin. The cultures were maintained at 37 °C in a humidified atmosphere containing 5% CO_2_.

### Transduction with miR-let-7a mimics/inhibitors and H19 siRNA

miR-let-7a mimic, inhibitor, and their respective negative controls (NC) were obtained from RiboBio Co., Ltd. (Guangzhou, China). Small interfering RNAs (siRNAs) targeting H19 (si-H19) and non-targeting control siRNAs were synthesized by RiboBio. The sequences used in HUVECs were as follow: si-H19-1: 5’-GGCCTTCCTGAACACCTTA-3’; si-H19-2: 5’-GACGTGACAAGCAGGACAT-3’; si-H19-3: 5’-CCTCTAGCTTGGAAATGAA-3’. The sequences used in MOVAS and RAW 264.7 were as follow: si-H19-1: 5’-GCAGAATGGCACATAGAAA-3’; si-H19-2: 5’-GGATCCAGCAAGAACAGAA-3’; si-H19-3: 5’-GCAGTCATCCAGCCTTCTT-3’. Cells were seeded in 6-well plates and transfected at 60-70% confluence using the riboFECT CP Transfection Reagent (RiboBio, Guangzhou, China) according to the manufacturer’s protocol. The final concentration of siRNAs was 50 nM, and appropriate doses were used for miRNA mimics/inhibitors. After 48 h of transduction, knockdown or overexpression efficiency was validated by quantitative real-time PCR (qPCR).

### Western blot analysis

HUVECs were seeded in 6-well plates and treated with oxLDL (100 μg/mL in ECM, Yiyuan Biotechnologies, Guangzhou, China) for 24 h at 37 °C under 5% CO_2_, while control cells received the vehicle (ECM alone). Total protein was extracted using RIPA lysis buffer supplemented with protease and phosphatase inhibitors. Lysates were centrifuged at 12,000 rpm for 15 min at 4 °C, and the protein concentration was determined using a BCA Protein Assay Kit (Beyotime Biotechnology, Shanghai, China). Equal amounts of protein (20 μg) were separated using SDS-PAGE and transferred onto PVDF membranes. Membranes were blocked with 5% non-fat milk in TBST (Tris-buffered saline with 0.1% Tween-20) and incubated overnight at 4 °C with primary antibodies against interleukin-6 (IL-6), interleukin-1β (IL-1β), tumor necrosis factor-alpha (TNF-α), ICAM-1, VCAM-1, N-cadherin (all from ABclonal, Wuhan, China), β-actin (Abcam, MA, USA), and GAPDH (Invitrogen, Waltham, USA). After washing with TBST, the membranes were incubated with horseradish peroxidase (HRP)-conjugated secondary antibodies for 1 h at room temperature. Protein bands were visualized using an enhanced chemiluminescence (ECL) reagent (Tanon, Shanghai, China) and imaged using an Amersham™ ImageQuant™ 800 system (Cytiva, Tokyo, Japan). Band intensities were quantified using the ImageJ software and normalized to the loading controls.

### RNA isolation and qPCR

Total RNA was extracted using TRIzol reagent (ABclonal, Wuhan, China). RNA purity and concentration were assessed using a NanoDrop™ 3000 spectrophotometer (Thermo Fisher Scientific, USA). 1μg of total RNA was reverse-transcribed into cDNA using a PrimeScript™ RT reagent kit (Sangon Biotech, Shanghai, China). QPCR was performed on a Real-Time PCR Detection System (Thermo Fisher Scientific, USA) using SYBR Green Master Mix (ABclonal). U6 was used as the internal reference for miR-let-7a, while β-actin served as the reference gene for mRNA targets.

### Statistical analysis

All experiments were independently repeated at least three times biologically, with three technical replicates for per sample. Data are presented as mean ± standard deviation (SD). Statistical comparisons between two groups were performed using an unpaired, two-tailed Student’s *t*-test, whereas comparisons among more than two groups were analyzed using one-way analysis of variance (ANOVA). All analyses were conducted using GraphPad Prism 8.0.2 (GraphPad Software, San Diego, CA, USA). Significance levels are denoted as follows: * represents P < 0.05, **represents P < 0.01, and ***represents P < 0.001.

## Result

### H19 is increased in aortae of AS mice and in oxLDL treated HUVECs

**Figure 1:**
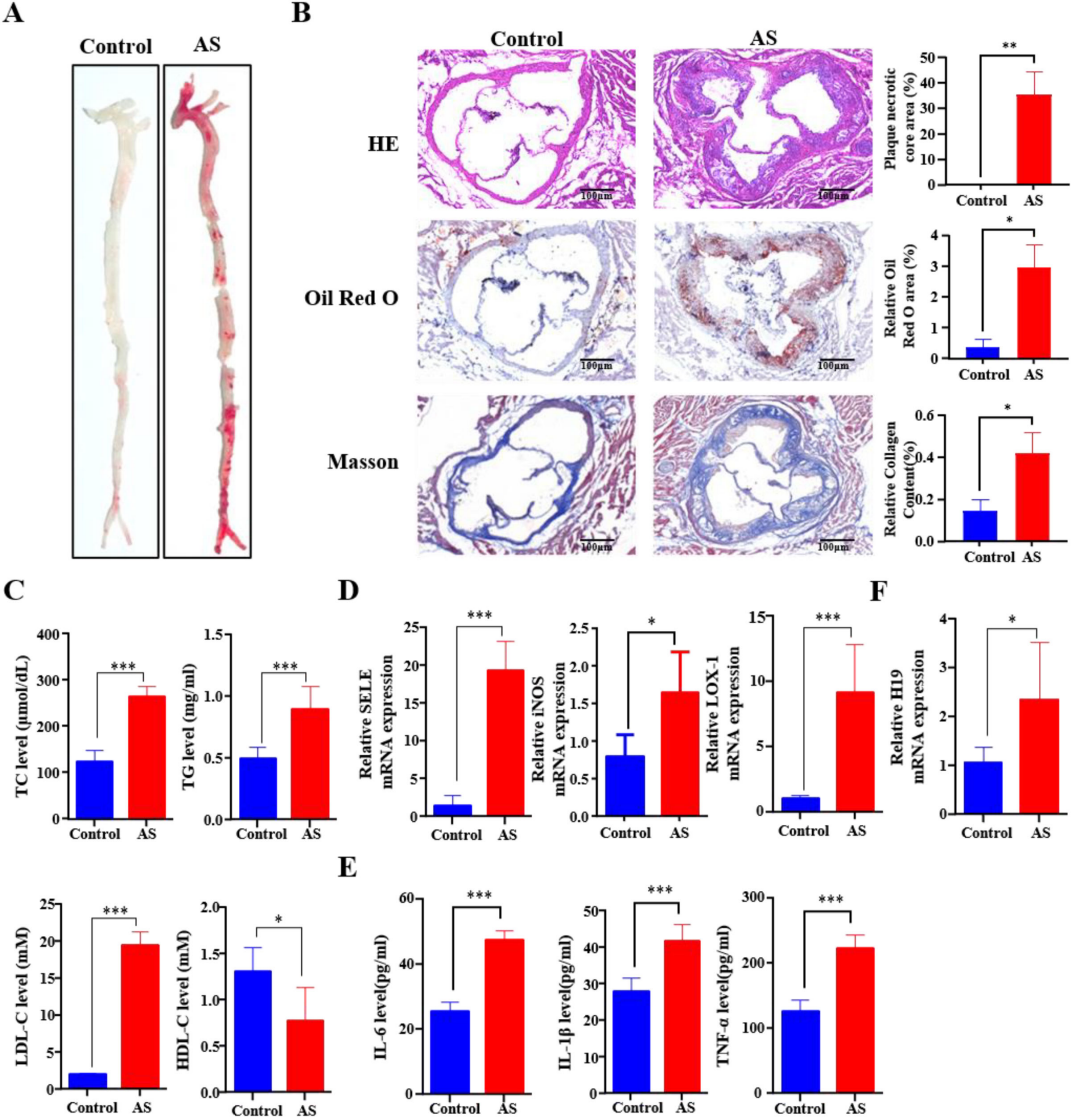
H19 is upregulated in the AS mouse model. **(A)** Oil red O staining of the entire aorta. **(B)** HE, oil red O, and masson staining of aorta root sections. The plaque necrotic core area, relative oil red O area, and relative collagen content were quantified using ImageJ software. **(C)** The serum level of TC, TG, LDL-C, and HDL-C. **(D)** SELE, iNOS, and LOX-1 mRNA levels of aorta, determined by qPCR. **(E)** The serum concentration of IL-6, IL-1β, and TNF-α. **(F)** H19 mRNA levels of aortic, determined by qPCR. *P<0.05, **P<0.01, ***P<0.001.

**Figure 2:**
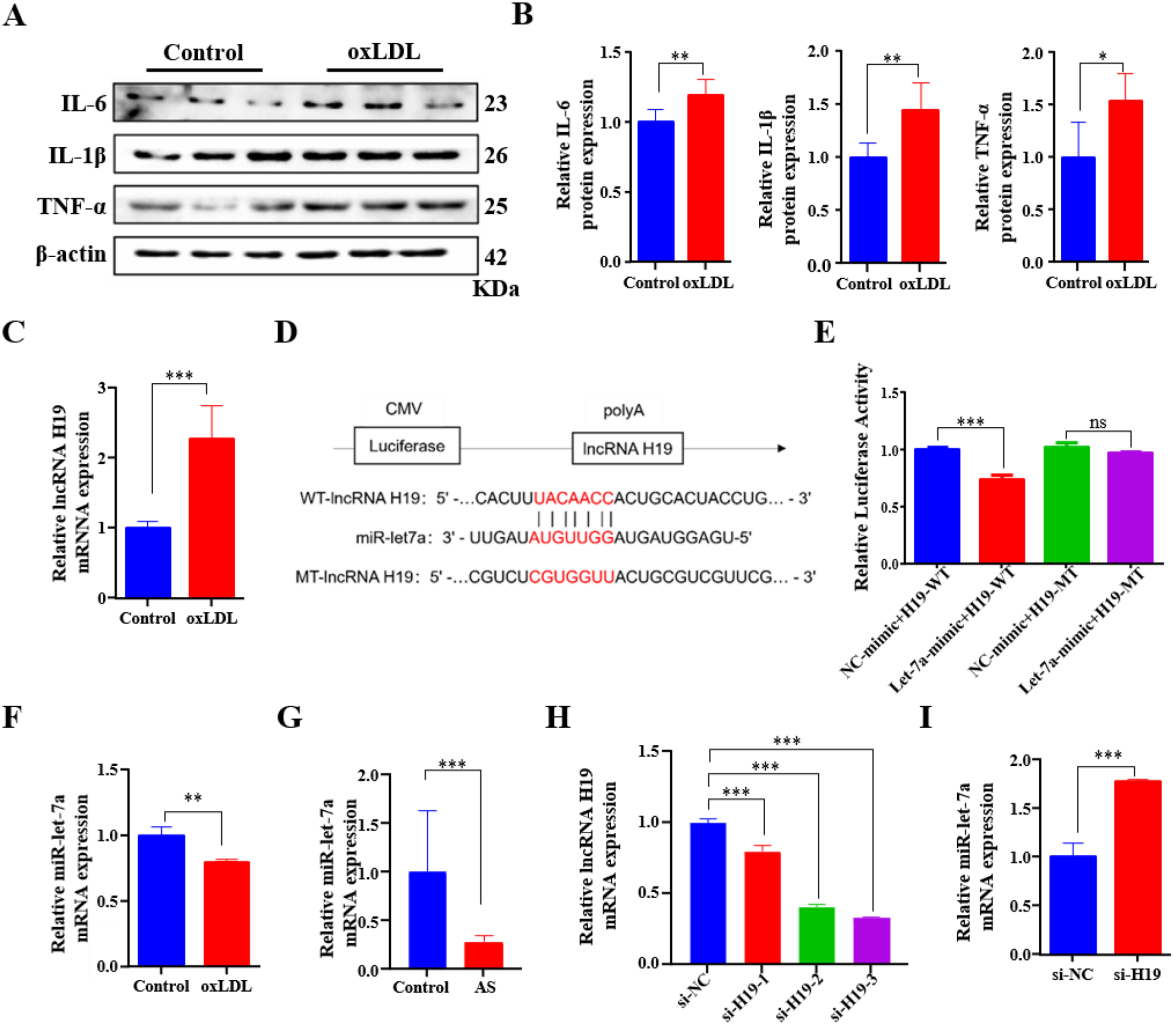
H19 alleviates the progression of AS via inhibited miR-let-7a expression. **(A, B)** Western blot analysis **(A)** and quantification **(B)** of IL-6, IL-1β, and TNF-α in HUVECs cell treated with oxLDL. **(C)** H19 mRNA levels in HUVECs cell following oxLDL treatment, determined by qPCR. **(D)** Schematic illustration of the predicted miR-let-7a binding site within the H19 sequence and the corresponding mutant construct. **(E)** HEK-293T cell were co-transfected with luciferase reporter plasmids containing either H19-WT or mutant H19-MT, together with miR-let-7a mimic or NC mimic. Luciferase activity was measured 48 h post- transduction. **(F, G)** miR-let-7a mRNA levels in oxLDL-treated HUVECs cell **(F)** and mouse aortic **(G)**, determined by qPCR. **(H)** H19 mRNA levels in HUVECs transfected with si-NC, si-H19-1, si-H19-2, or si-H19-3, determined by qPCR. **(I)** miR-let-7a mRNA levels in HUVECs cell transfected with si-NC and si-H19-3, determined by qPCR. *P<0.05, **P<0.01, ***P<0.001.

*ApoE*^-/-^ mice are a widely accepted experimental model for AS (Zadelaar et al., 2007). After feeding *ApoE*^-/-^ mice a high-fat diet for 10 weeks, successful establishment of the AS model was confirmed by pathological evaluation. To assess AS lesion formation, Oil Red O staining was performed to visualize the lipid-rich plaques. Compared to the control group, the AS model group exhibited substantial lipid accumulation throughout most of the aortic region (**Figure 1A**). Pathological changes in the aortic roots were also evaluated. HE staining revealed a significantly larger necrotic core area in plaques from the AS model group (**Figure 1B**). Oil Red O staining showed increased lipid deposition in the intima of the aortic root, and Masson’s trichrome staining demonstrated pronounced fibrosis (**Figure 1B**). These findings collectively confirmed the successful induction of the AS mouse model. Given that dyslipidemia is a hallmark of AS, relevant serum lipid parameters were also assessed. The results showed that TC, LDL-C, and TG levels were significantly elevated, whereas HDL-C levels were markedly reduced in the AS group compared to the control group (**Figure 1C**), confirming that the AS mouse model recapitulates the characteristic dyslipidemia observed in human AS. Furthermore, expression of AS related proteins, including E-selectin (SELE), inducible nitric oxide synthase (iNOS), and lectin-like oxidized LDL receptor-1 (LOX-1) were significantly upregulated in aortic tissues from the AS group relative to the control group (**Figure 1D**), indicating enhanced endothelial activation, oxidative stress, and uptake of oxidized LDL, all of which are key molecular features of AS plaque development and progression. Additionally, the serum levels of the pro-inflammatory cytokines IL-6, IL-1β, and TNF-α were markedly increased in the AS group (**Figure 1E**), indicating vascular endothelial dysfunction, systemic inflammation, and active progression of AS lesions. Finally, H19 expression was examined, and the results showed that H19 levels were significantly elevated in the AS group (**Figure 1F**). To establish an *in vitro* model of AS, HUVECs were treated with oxLDL. This treatment significantly increased the protein levels of IL-1β, IL-6, and TNF-α (**Figures 2A, B**). Consistent with the *in vivo* findings, H19 expression was markedly increased in the AS cell model (**Figure 2C**). Taken together, these results demonstrate that H19 is highly expressed in both the mouse and cell models of AS.

### H19 alleviates the progression of AS via inhibiting miR-let-7a expression

Accumulating evidence suggests that lncRNAs can function as ceRNAs or “miRNA sponges”, thereby sequestering miRNAs and reducing their binding to target mRNAs (Tay et al., 2014). Given the important role of miR-let-7a in mediating the effects of H19 (Kallen et al., 2013), the relationship between H19 and miR-let-7a in AS was examined. The wild-type H19 sequence containing the predicted miR-let-7a binding site and a mutant version in which the binding site was disrupted were cloned downstream of the luciferase gene in a reporter plasmid for use in luciferase assays (**Figure 2D**). Luciferase assay results showed that co-transduction with a miR-let-7a mimic significantly suppressed luciferase activity in cells expressing the wild-type reporter, whereas this suppression was abolished in the mutant construct (**Figure 2E**). Moreover, miR-let-7a expression was downregulated in both the oxLDL-induced AS cell model (**Figure 2F**) and *ApoE*^-/-^ mouse AS model (**Figure 2G**). Furthermore, siRNA was used to knock down H19 expression in HUVECs. Among the tested siRNAs, si-H19–3 exhibited the highest knockdown efficiency and was selected for subsequent experiments (**Figure 2H**). Consistent with the ceRNA hypothesis, H19 knockdown using si-H19–3 further increased miR-let-7a levels in HUVECs (**Figure 2G**). Taken together, these data suggest that H19 promotes AS progression by suppressing miR-let-7a activity, whereas restoration of miR-let-7a mitigates oxLDL-induced damage in EC.

### H19 sponged miR-let-7a to increase ITGB3 expression

**Figure 3:**
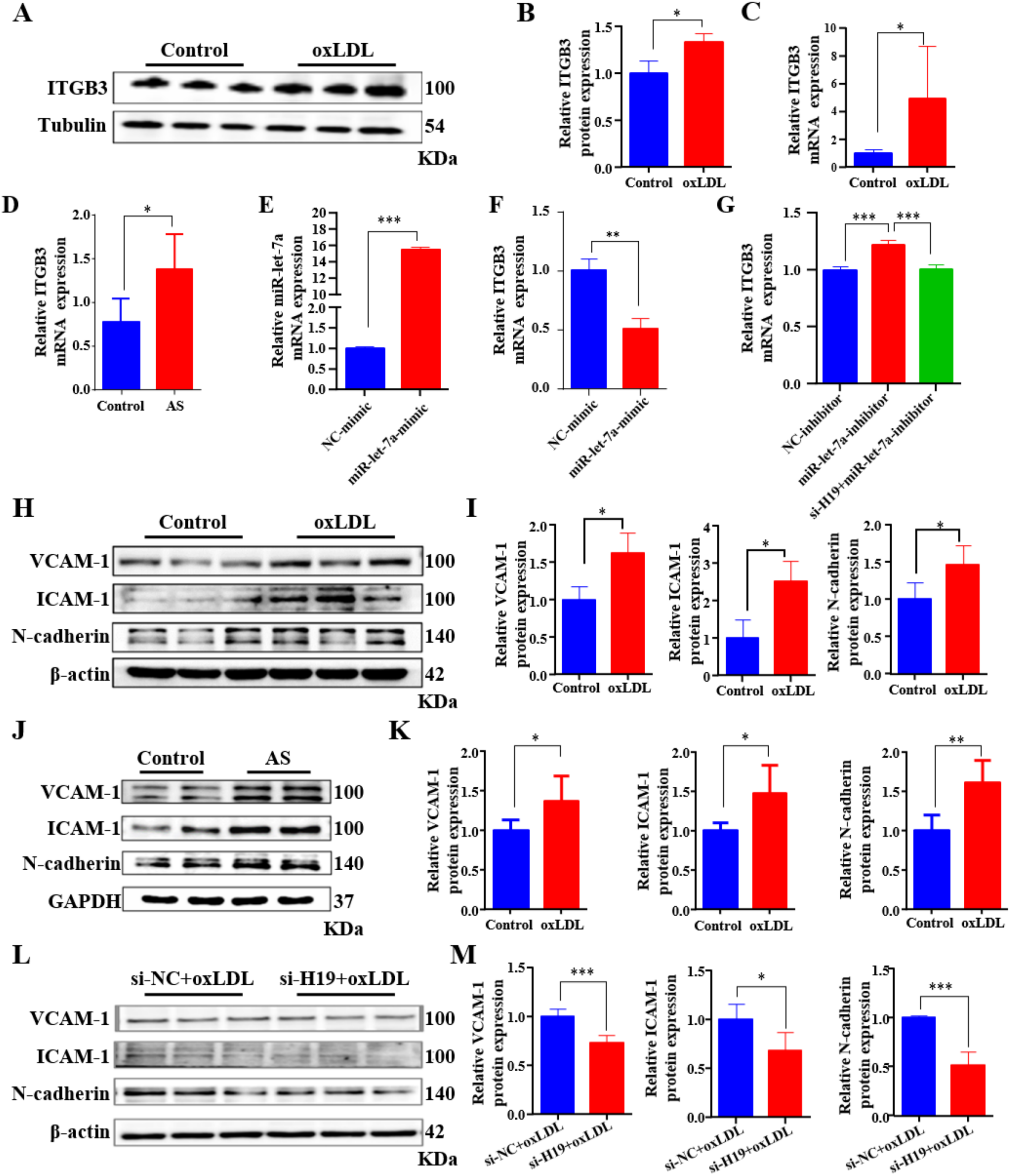
H19 sponged miR-let-7a to increase ITGB3 expression. **(A, B)** Western blot analysis **(A)** and quantification **(B)** of ITGB3 in oxLDL-treated HUVECs. **(C)** ITGB3 mRNA levels in oxLDL-treated HUVECs cell, determined by qPCR. **(D)** ITGB3 mRNA levels in aortic, determined by qPCR. **(E, F)** miR-let-7a **(E)** and ITGB3 **(F)** mRNA levels in HUVECs transfected with NC-mimic and miR-let-7a-mimic, determined by qPCR. **(G)** ITGB3 mRNA levels in HUVECs transfected with NC-inhibitor, miR-let-7a-inhibitor or si-H19+miR-let-7a-inhibitor, determined by qPCR. **(H, I)** Western blot analysis **(H)** and quantification **(I)** of VCAM-1, ICAM-1, and N-cadherin in oxLDL-treated HUVECs. **(J, K)** Western blot analysis **(J)** and quantification **(K)** of VCAM-1, ICAM-1, and N-cadherin in aortic. **(L, M)** Western blot analysis **(L)** and quantification **(M)** of VCAM-1, ICAM-1, and N-cadherin in oxLDL-treated HUVECs transfected with si-NC and si-H19. *P<0.05, **P<0.01, ***P<0.001.

ITGB3 has been identified as a potential target of miR-let-7a (Cheong et al., 2012; Zhao et al., 2014). To validate this, ITGB3 expression was examined in both *in vitro* and *in vivo* AS models. The results showed that *in vitro* oxLDL treatment significantly increased ITGB3 expression at both the mRNA and protein levels (**Figures 3A–D**), suggesting transcriptional upregulation. To determine whether miR-let-7a directly regulates the expression of ITGB3, HUVECs were transfected with miR-let-7a mimic (**Figure 3E**). The results showed that overexpression of miR-let-7a markedly suppressed ITGB3 mRNA levels (**Figure 3F**), confirming its inhibitory role. Given the existence of a ceRNA network involving H19, miR-let-7a, and ITGB3, the requirement of miR-let-7a for H19-mediated ITGB3 regulation was investigated. HUVECs were co-transfected with si-H19–3 and miR-let-7a inhibitor. Knockdown of H19 reversed the suppressive effect of miR-let-7a on ITGB3 expression (**Figure 3G**), indicating that H19 promotes ITGB3 expression by acting as a ceRNA that sequesters miR-let-7a. Inflammation plays a critical role in AS progression (Libby, 2012). Therefore, the relationship between the H19/miR-let-7a/ITGB3 axis and inflammatory responses was investigated. The expression of key adhesion factors, including VCAM-1, ICAM-1, and N-cadherin, was significantly elevated in both the AS cell model (**Figures 3H, I**) and the AS mouse model (**Figures 3J, K**). Silencing of H19 in HUVECs abolished this upregulation (**Figures 3L, M**), demonstrating that H19 drives endothelial inflammation through the enhanced expression of these adhesion proteins. Collectively, these findings indicate that H19 contributes to AS lesion development by functioning as a ceRNA for miR-let-7a to derepress ITGB3 expression and promote vascular inflammation.

### *In vivo* transduction with AAV-sh-H19 alleviated the progression of AS

**Figure 4:**
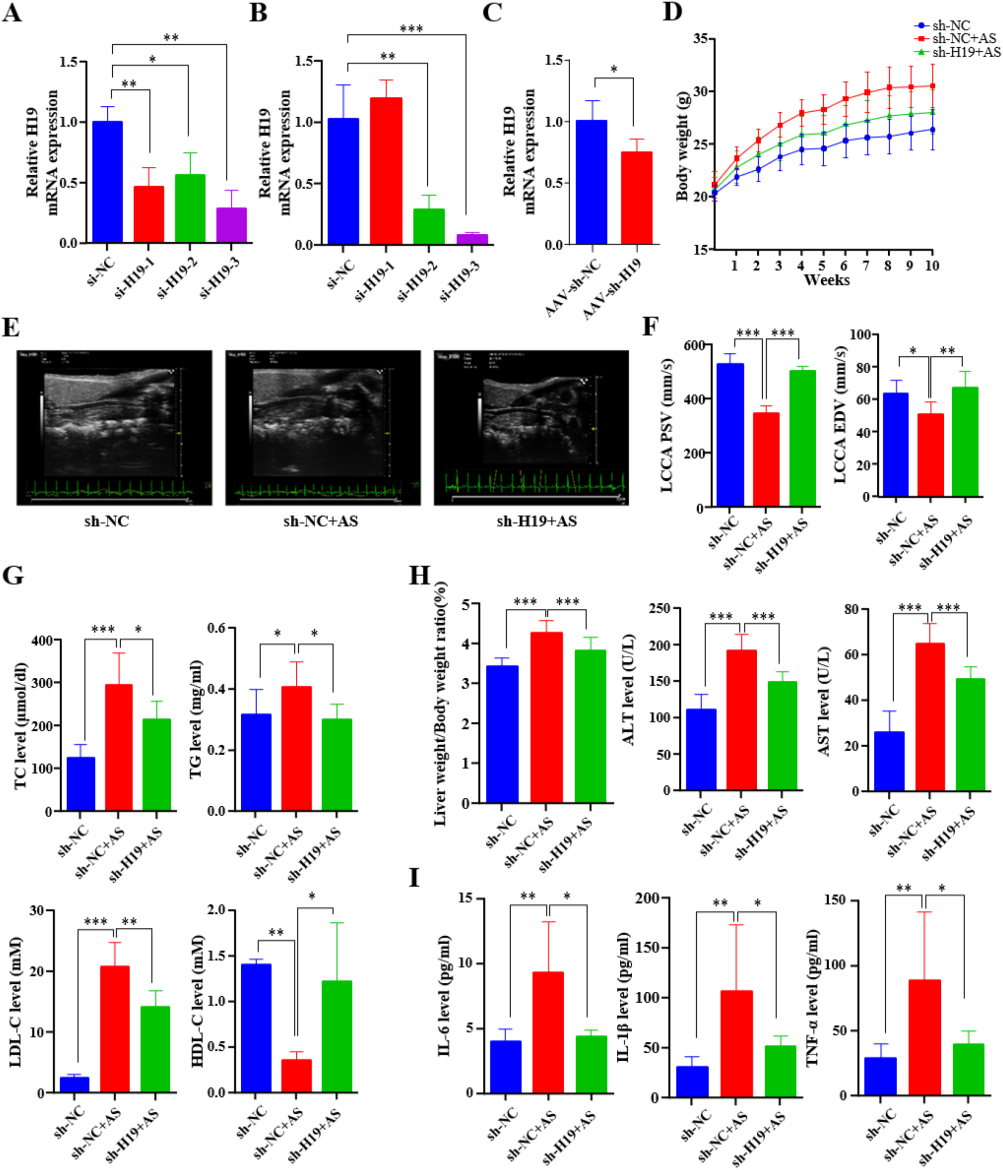
AAV-sh-H19 attenuates AS progression. **(A, B)** H19 mRNA levels in MOVAS **(A)** and RAW 264.7 **(B)** transfected with si-NC, si-H19-1, si-H19-2, or si-H19-3, determined by qPCR. **(C)** H19 mRNA levels in aortic tissues of *ApoE^-/-^* mice following tail vein injection of AAV-sh-NC and AAV-sh-H19, determined by qPCR. **(D)** Body weight monitored throughout the experiment period. **(E)** Ultrasound image of the LCCA in each group. **(F)** PSV and EDV of the LCCA **(G)** The serum level of TC, TG, LDL-C, and HDL-C in the different group. **(H)** Liver to Body weight ratio and serum level of ALT and AST. **(I)** Serum concentrations of IL-6, IL-1β, and TNF-α. *P<0.05, **P<0.01, ***P<0.001.

**Figure 5:**
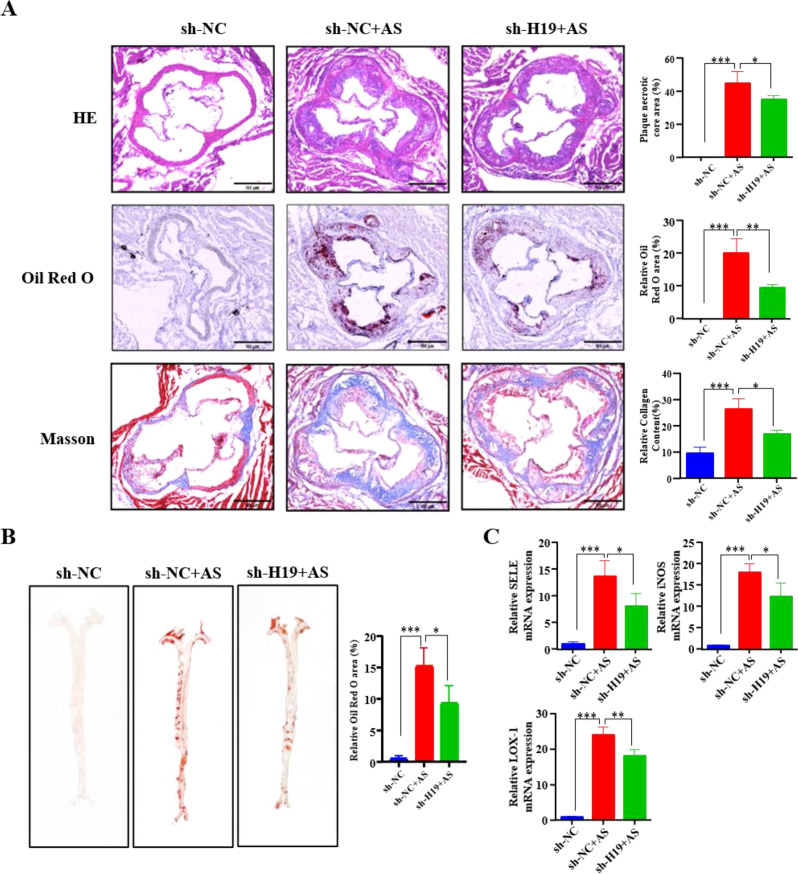
AAV-sh-H19 alleviated the vasculopathy of AS. **(A)** HE, oil red O, and masson staining of aorta root sections. The plaque necrotic core area, relative oil red O area, and relative collagen content were quantified using ImageJ software. **(B)** Oil red O staining of the entire aorta. The relative oil red O area was quantified using ImageJ software. **(C)** SELE, iNOS, and LOX-1 mRNA levels in aortic, determined by qPCR. *P<0.05, **P<0.01, ***P<0.001.

To evaluate the role of H19 in the *in vivo* AS model, AAV-sh-H19 was delivered via tail vein injection into *ApoE*^-/-^ mice, which were subsequently maintained on a high-fat diet for 10 weeks. Prior to *in vivo* experiments, H19 knockdown efficiency was validated *in vitro*. MOVAS and RAW 264.7 cells were transfected with three distinct siRNAs targeting H19, and si-H19–3 demonstrated the highest silencing efficacy. Therefore, si-H19–3 was selected for subsequent experiments (**Figures 4A, B**). Based on this sequence, a shRNA (sh-H19) was designed and packaged into AAV for systemic delivery. qPCR analysis of aortic tissues confirmed that H19 expression was significantly reduced in the AAV-sh-H19 group compared to that in the AAV-sh-NC group (**Figure 4C**). During the 10-week high-fat diet, H19 knockdown significantly attenuated body weight gain (**Figure 4D**), suggesting a role for H19 in the regulation of metabolism. Vascular function was assessed using a high-frequency ultrasound. The results showed that AS induction markedly reduced both peak systolic velocity (PSV) and end-diastolic velocity (EDV) in the left common carotid artery (LCCA), indicating impaired hemodynamics in the LCCA. Notably, H19 silencing partially restored these parameters, demonstrating improved vascular function (**Figures 4E, F**). Moreover, H19 knockdown ameliorated dyslipidemia, as evidenced by significantly lower serum TC, LDL-C, and TG levels, along with a concomitant increase in HDL-C (**Figure 4G**). Given the frequent co-occurrence of hepatic dysfunction in high-fat diet-induced AS models, we evaluated liver-related parameters. The liver-to-body weight ratio was markedly elevated in the AS control group, indicating hepatomegaly, whereas H19 knockdown significantly mitigated this increase (**Figure 4H**). Consistently, serum levels of ALT and AST, which are well-established biomarkers of hepatocellular injury, were substantially elevated in AS mice but reduced upon H19 knockdown (**Figure 4H**). These results suggest that H19 contributes not only to vascular pathology but also to systemic metabolic and hepatic disturbances in AS patients. In addition, H19 knockdown significantly suppressed systemic inflammation, as reflected by decreased serum levels of the pro-inflammatory cytokines IL-6, IL-1β, and TNF-α (**Figure 4I**). Furthermore, Histopathological analyses of the aortic roots further corroborated the protective effects of H19 inhibition. HE staining revealed a smaller necrotic core area, Oil Red O staining showed reduced lipid deposition, and Masson staining indicated diminished fibrosis in the sh-H19+AS group compared with the sh-NC+AS group (**Figures 5A, B**). At the molecular level, the expression of key AS-related markers, including SELE, iNOS, and LOX-1, was markedly downregulated in aortic tissues following H19 silencing (**Figure 5C**). Collectively, these findings demonstrate that H19 plays a pivotal proatherogenic role *in vivo*. Its knockdown alleviates AS progression by improving lipid metabolism, attenuating hepatic injury, suppressing inflammation, reducing endothelial activation, and restoring vascular hemodynamic function.

## Discussion

AS is a chronic and progressive inflammatory disorder of the arterial wall that underlies the majority of cardiovascular diseases. Driven by a complex interplay of metabolic, hemodynamic, and immunological factors, AS is characterized by the subendothelial accumulation of modified lipoproteins, particularly oxLDL, along with the recruitment and infiltration of immune cells, VSMC migration from the media to the intima, and extracellular matrix remodeling. Over time, these processes culminate in the formation of plaques featuring a lipid-rich necrotic core covered by a fibrous cap composed of VSMCs and collagen (Morrison et al., 2023). In this study, we demonstrated that H19 functions as a ceRNA by acting as a molecular sponge for miR-let-7a. Specifically, by sequestering miR-let-7a, H19 relieves its post-transcriptional repression of ITGB3, leading to elevated ITGB3 protein levels. Consequently, increased ITGB3 expression enhances the production of cell adhesion molecules, thereby promoting endothelial activation and exacerbating vascular inflammation.

Despite decades of research, the precise molecular pathways governing the initiation and progression of AS remain incompletely understood. However, recent advances in non-coding RNA biology have revealed that lncRNAs, transcripts longer than 200 nucleotides with limited protein-coding potential, serve as pivotal regulators of vascular homeostasis and inflammation (Ma et al., 2023). Among the numerous lncRNAs implicated in cardiovascular pathology, H19 stands out because of its high degree of evolutionary conservation across species and its consistent dysregulation in atherogenic contexts (Hurst and Smith, 1999). Originally identified as an imprinted gene involved in embryonic development, H19 has since been recognized as a potent modulator of cellular proliferation, differentiation, and inflammatory signaling in adult tissues. Accumulating evidence now positions H19 as a key driver of AS pathogenesis (Shi et al., 2020). To investigate the functional role of H19 in AS, this study employed two complementary experimental models widely accepted in the field, an *in vivo* model using *ApoE*^-/-^ mice fed a high-fat diet to induce hyperlipidemia and plaque formation, and an *in vitro* model based on HUVECs stimulated with oxLDL to mimic early endothelial dysfunction. Both models effectively recapitulate critical aspects of human AS and have been extensively validated for mechanistic studies. Consistent with clinical observations, we found that H19 expression was significantly upregulated in the aortic tissues of *ApoE^-/-^* mice and in oxLDL-treated HUVECs.

The pathogenesis of AS typically originates from endothelial dysfunction, a condition precipitated by traditional risk factors such as hypertension, dyslipidemia, diabetes mellitus, and smoking. These factors impair nitric oxide bioavailability, increase oxidative stress, and disrupt the integrity of the endothelial barrier. Under such conditions, circulating LDL particles infiltrate the arterial intima, where they undergo oxidative modification by reactive oxygen species and enzymatic activity to form oxLDL. OxLDL acts as a damage-associated molecular pattern, activating pattern recognition receptors on EC and inducing the transcriptional upregulation of adhesion molecules, notably VCAM-1 and ICAM-1. These surface proteins facilitate firm adhesion and subsequent transendothelial migration of circulating monocytes into the subendothelial space (Sluiter et al., 2021). Once inside the vessel wall, monocytes differentiate into macrophages under the influence of colony-stimulating factors. Through scavenger receptor-mediated uptake of oxLDL, these macrophages become engorged with cholesterol esters, transforming into foam cells, which are the defining cellular components of early fatty streaks (Yu et al., 2013). As the lesion evolves, VSMCs undergo phenotypic modulation from a contractile to a synthetic state, migrate across the internal elastic lamina into the intima, and secrete abundant ECM components, particularly type I and III collagen. This process forms a stabilizing fibrous cap that encapsulates the necrotic core, which consists of dead foam cells, extracellular lipids, and cellular debris (Alonso-Herranz et al., 2023). However, in advanced or unstable plaques, persistent inflammation driven by macrophage-derived proteases, such as matrix metalloproteinases, degrades the collagenous scaffold. This degradation thins the fibrous cap, predisposing the plaque to rupture.

Emerging research underscores the multifaceted regulatory roles of lncRNAs in the pathogenesis of AS. Beyond H19, numerous lncRNAs have been shown to modulate lipid metabolism, inflammasome activation, endothelial permeability, VSMC plasticity, and macrophage polarization (Arslan et al., 2017). Notably, H19 is markedly overexpressed in human AS plaques compared to healthy coronary arteries (Han et al., 1996), and its plasma concentration is significantly elevated in patients with established AS relative to healthy individuals (Pan, 2017), suggesting its potential as both a biomarker and a therapeutic target. Previous studies have demonstrated that H19 exerts its biological effects primarily by acting as a molecular sponge for various miRNAs (Busscher et al., 2022). For instance, H19 promotes intracellular lipid accumulation in macrophages by sponging miR-146a-5p, thereby derepressing angiopoietin-like protein 4 (ANGPTL4), a key regulator of lipoprotein lipase activity (Huang et al., 2021). Furthermore, H19 has been shown to alleviate inflammation by sponging miR-20a-5p, which leads to the activation of the HDAC4 axis (Yang et al., 2022). In the context of VSMC, H19 regulates foam cell formation by inhibiting miR-107, subsequently activating the CD40/CD40L signaling pathway, a well-established pro-inflammatory mechanism in AS (Zhang et al., 2025). Regarding the miR-let-7a axis, H19 is known to upregulate cyclin D1 via miR-let-7a sequestration, thereby inhibiting the abnormal proliferation of vascular smooth muscle cells (Sun et al., 2019). However, the relationship between the H19/miR-let-7a axis and EC has not been described here. In another study, H19 was shown to upregulate periostin through miR-let-7a, influencing cell adhesion (Cao et al., 2019). However, that study lacked *in vivo* validation. Given that ECs are the primary cell type affected in AS (Gimbrone and García-Cardeña, 2016), this study focuses on the influence of the H19/miR-let-7a axis on EC and validates the findings through system *in vivo* experiments.

ITGB3 is essential for cell adhesion to multiple ECM substrates, including fibrinogen, fibronectin, and collagen, and its expression correlates with enhanced endothelial-leukocyte interactions (Ni et al., 2015; Li et al., 2024). Previous studies have demonstrated that miR-let-7a modulates blastocyst implantation in both mice and humans by targeting ITGB3 (Cheong et al., 2012). Furthermore, miR-let-7a has been shown to regulate migration and invasion in human non-small cell lung cancer through the same ITGB3 axis (Zhao et al., 2014). Although the direct relationship between ITGB3 and atherosclerosis (AS) remains underexplored, inflammatory cell infiltration is critically dependent on cell adhesion mechanisms. Given this mechanistic link, ITGB3 was investigated in the present study to elucidate the specific functional role of the miR-let-7a/ITGB3 axis in the context of AS. Moreover, increased expression of VCAM-1, ICAM-1, and N-cadherin was observed in both *in vivo* and *in vitro* AS models, and all were significantly attenuated following H19 knockdown. N-cadherin serves as a hallmark marker of endothelial-to-mesenchymal transition (EndoMT). Its upregulation signifies a loss of endothelial identity and tight junction integrity, a process that not only facilitates greater oxLDL penetration into the intima but also amplifies leukocyte adhesion and transmigration. These events collectively accelerate plaque initiation and drive inflammatory propagation (Liu et al., 2024). Hence, by bridging the gap between H19/miR-let-7a/ITGB3 axis and cellular adhesion, this study clarifies the role of this pathway in the pathogenesis of AS and provides compelling evidence that targeting it could serve as a novel therapeutic strategy to mitigate endothelial dysfunction and halt the progression of atherosclerotic lesions.

Chronic inflammation is a central feature of AS pathophysiology, playing a decisive role in both plaque progression and destabilization (Ajoolabady et al., 2024). While stable plaques are characterized by a state of chronic low-grade inflammation, active inflammation driven by cytokine networks, inflammasome activation, and immune cell crosstalk can critically destabilize plaque architecture (Nahrendorf, 2018). The initial trigger of this cascade is the accumulation and oxidation of LDL within the subendothelial space. OxLDL activates local endothelial and immune cells and propagates systemic inflammation by stimulating bone marrow hematopoiesis, thereby releasing monocytes into circulation. These monocytes are subsequently recruited to nascent lesions via chemotactic gradients, predominantly mediated by monocyte chemoattractant protein-1 (MCP-1) (Pirillo et al., 2013). Upon entering the intima, these cells differentiate into macrophages or dendritic cells, both of which perpetuate local inflammation. Initially, macrophages attempt to clear the lipid load through the phagocytosis of oxLDL. However, when lipid influx overwhelms efflux capacity, cholesterol crystals accumulate, driving the transformation of macrophages into foam cells (Moore and Tabas, 2011). These foam cells lose their protective function and secrete pro-inflammatory cytokines and chemokines, further driving plaque inflammation and growth (Gui et al., 2022). As the plaque expands, impaired clearance of apoptotic cells leads to the accumulation of cellular debris and expansion of the necrotic core, exacerbating inflammation and compromising plaque stability (Adkar and Leeper, 2024). It is important to acknowledge a limitation regarding our *in vivo* experimental design. The AAV vectors were administered systemically, and given the hepatic natural tropism for AAV, there is a possibility that the virus directly transduced hepatocytes, leading to hepatic H19 knockdown independent of vascular effects. While our data on reduced systemic inflammatory markers and endothelial adhesion molecules strongly supports a primary mechanism mediated by vascular protection, we cannot entirely exclude the contribution of direct hepatic modulation to the observed phenotypic improvements. In this context, the present study demonstrated that H19 act as a critical exacerbator of vascular inflammation in AS. Knockdown of H19 significantly reduced the expression of adhesion molecules and attenuated inflammatory markers. These finding suggest that H19 promotes endothelial activation and leukocyte recruitment through the upregulation of ITGB3 and related adhesion proteins.

## Conclusion

In conclusion, this study elucidates a novel pathogenic mechanism of the H19 in the context of AS. We demonstrate that H19 functions as a ceRNA by sponging miR-let-7a, which leads to the upregulation of ITGB3. This regulatory axis results in enhanced production of endothelial adhesion molecules and the sustain of vascular inflammation. Significantly, this work advances the field by providing the first experimental validation of ITGB3’s functional role in AS pathogenesis, supported by systematic *in vivo* evidence using ApoE-/- mouse models. These findings establish H19 as a critical upstream regulator of early atherogenic events, effectively bridging non-coding RNA biology with the molecular basis of endothelial dysfunction. Given its marked upregulation in human AS plaques and its demonstrable pathogenic effects in experimental models, H19 represents a promising therapeutic target. Consequently, future strategies aimed at inhibiting H19 expression or disrupting its interaction with miR-let-7a may offer innovative approaches for the prevention and treatment of AS-related cardiovascular diseases.

## Data Availability

The original contributions presented in the study are included in the article/supplementary material. Further inquiries can be directed to the corresponding author/s.
